# The Early Diagnosis of Pregnancy*Abstract of a paper read before the American Medical Association, at St. Louis, May, 1886.

**Published:** 1886-07

**Authors:** E. S. McKee

**Affiliations:** Cincinnati, O.


### * The Early Diagnosis of Pregnancy.

By E. S. McKee, M. D., Cincinnati, O.

The opprobrium to obstetrics is our inability to make a prompt and positive diagnosis of pregnancy in the earlier months. How often we are confronted by one of our female

* Abstract of a paper read before the American Medical Association, at St. Louis, May, 1886.

patients with the query, "Am I pregnant?" Many a woman imagines that her doctor can look at her, and tell her whether she is pregnant. How often great things depend upon our decision. Our patient may wish to take a long and long-looked-for journey, but will defer, postpone, or abandon it if she is pregnant; or other affairs of import to the question of operative interference may depend on our answer. In fact, fortunes, empires and lives hang on our ultimatum. That she fears she is to be a mother though not a wife, thinks her case of no less importance.

It is in the first months of gestation that the physician needs reliable evidence; hence, we should endeavor to add to the means already at our disposal, any symptoms indicative of the presence or absence of pregnancy.

It is with this in view that your essayist will endeavor to rehearse the symptoms, confining himself to those of the first trimeserium. Those symptoms well known will receive but passing comment. Those newer, and, as yet, not fully established in our text-books, shall have more attention.

First, in the natural order of things, we consider the signs which may occur at the time of conception. Aristotle taught that a woman had conceived if there was no return of semen after sexual intercourse, and if the penis was unusually dry when withdrawn. Such is also the universal opinion of shepherds. Hippocrates said that the eyes sank in their sockets; Demeritus, that the neck swelled. These, however, are of little value. Probably the only one of any worth is the peculiar voluptuous sensation, and more general erethism experienced by some females on fruitful intercourse.

After discussing in detail the well-known signs of the first three months, and others not quite so well known, and lamenting the fallibility of them all, we turn our attention to the sign of Hegar, as follows:

To Hegar, of Freiburg, we are indebted for the new sign of great promise which bears his name.

This sign consists of an unusual resilience, compressibility, softness, bogginess, yielding and thinning of the lower uterine segment; that is, the section immediately above the insertion of the cervic supra-uterine. This condition is not alone present when the remainder of the body, as is often the case, is firm and hard, but also quite prominent when this is soft and elastic. The shape assumed is fan-like, or that of a balloon, more than the usual pear shape; it has also been termed an old-fashioned, ear-bellied jug. This enlargement is especially marked antero-posteriorly. The change is most apparent at the middle portion of the lower segment, and in the median line, sides of the organ being much firmer and more resisting.

Compes makes the examination as follows: The thumb is introduced into the vagina until it reaches the cervix, and the index into the rectum until it reaches beyond the ligamenta sacro-uterina; the other hand is placed over the abdomen, immediately above the symphysis, and pressed down towards the finger in the rectum. The rectal finger explores the cervix and the lower uterine segment in all its parts, and, lastly, the higher parts of the uterus. The examination is facilitated by pulling down the uterus with a vulsellum forceps, and evacuating the bladder and rectum.

While this is undoubtedly a very thorough mode of examination, it is repulsive both to the patient and physician, as well as a difficult and hazardous procedure. It is certainly possible, in a great majority of cases, to make out all that is necessary with the finger in one of the culs-de-sac and the other hand externally. In urgent cases, where this does not satisfy the physician, it would be quite proper to make an examination as above described.

Compes has examined a number of women, found the sign present, then put them under an anæsthetic, and still found it present. He says the softened and enlarged uterine segment above the cervix has often been mistaken for a tumor, and that, in fact, laparotomy has been performed under this delusion.

There are two states which may simulate this condition, viz.: distended bladder, and the uterus distended with menstrual blood. A distended bladder can, and should be, evacuated. An imperforate hymen or vagina, or the history of the case would soon dispel the other question. Hyperplasia would show increased density. Sub-involution increases the longitudinal as well as the transverse diameters. The obstructed circulation from an anteflexed uterus does not impart that feeling of resiliency and compressibility. In marked retroversion it is more difficult to palpate the corpus uteri, and the sign may fail. Here, also, it is proper to examine per rectum.

Dr. Reinl, formerly assistant to Hegar, has reported six cases in the literature, and by letter he tells extended experience as follows: "Among twenty-two cases, I missed this sign but twice, and found it earliest in the fifth week of pregnancy, a primi-para, and a very near relation."

Dr. Compes, present assistant to Hegar, reports eight cases.

Dr. E. H. Grandin, of New York, has reported twelve cases in a letter to me; since this report he says: "Since the writing of my paper, I have had six additional cases, all corroborated, and one of these a case of retroversion. Personally, therefore, I record myself as being able to make the diagnosis prior to the sixth week, by Hegar's sign alone."

My own experience has been so recent, that most of my cases have not yet had time to be corroborated. I will mention but two, one in which the sign was absent, one in which it was present.

Case I. Mrs. B., a widow, æt. 37, came to me March 20, 1886. She acknowledged the opportunity, and feared herself pregnant, not having seen her menses for twelve weeks. I examined her for Hegar's sign, but failed to find it. I told her I did not think her pregnant, gave her tincture ferri chloridi, and asked her to return in a few days. She returned three times, each time expressing great fears of pregnancy. Each time I examined her, failed to find Hegar's sign, assured her she was not pregnant and continued the iron. April 1st, the menses re-appeared and were normal in amount and duration.

Case II. Mrs. R., a young married woman, æt. 20, a delicate blonde, the mother of one child, æt. 2 years. She had been absent from her husband four months, visiting her parents at Washington, D. C. She last had her menses January 15th, continuing five days; normal in amount and conduct. She returned to Cincinnati, and her husband, February 9th. March 5th, Dr. Ransohoff was called, and finding the case gynæcological, referred her to me. She had not had a return of her menses since the middle of January. The nature of her case required a digital and specular examination, once, twice, or thrice weekly. March 10th, she was slightly sick at the stomach; this had not occurred before, and did not recur, nor any other sign indicating pregnancy, besides the cessation of the menses, before the sixth week after her return to her husband. During the sixth week I made three careful vaginal examinations, and at each one was sure I found Hegar's sign present. I assured the patient that I was quite confirmed in the belief that I had frequently expressed to her, viz.: that she was pregnant.

On the 30th of March, she complained of not feeling well. On the morning of March 31st, the forty-eighth day after her return, she passed a large quantity of blood, and a membrane, which she saved and showed me. This proved to be the major portion of an ovum, the remainder of which was found within the vagina.

